# Developing a model to facilitate the preparedness of inexperienced research professionals for a joint research journey in nursing education institutions

**DOI:** 10.4102/curationis.v48i1.2725

**Published:** 2025-12-20

**Authors:** Masenyani O. Mbombi, Sindiwe James, Esmeralda Ricks

**Affiliations:** 1 Department of Nursing Sciences, Faculty of Health Sciences, University of Limpopo, Polokwane, South Africa; 2 Department of Nursing Science, Faculty of Health Sciences, Nelson Mandela University, Gqeberha, South Africa

**Keywords:** facilitating preparedness, post-graduate research nursing students, inexperienced research supervisors, resilience, research support

## Abstract

**Background:**

The post-graduate completion period plays a significant role in individual career development and funding research activities in Nursing Education institutions (NEIs). Nursing Education institutions must produce more post-graduate research nursing students (PGRNSs) and supervisors to sustain and enhance research funding opportunities. Nevertheless, there is a dearth of information and documentation on strategies to improve supervisors’ and students’ preparedness for collaboration throughout the post-graduate study.

**Objectives:**

The study aims to describe the process followed when developing a model to facilitate the preparedness of post-graduate students and research supervisors for a joint research journey.

**Method:**

The study utilised a theory-generating, qualitative, exploratory, descriptive and contextual design, grounded by Chinn and Kramer’s four stages of model development to achieve the aim. The four steps guided the conceptual framework for the development of the model. Data collected from 16 postgraduate students and 12 research supervisors were analysed with content thematic analysis.

**Results:**

Four themes were generated; perceptions regarding postgraduate supervision provided or received, perceptions regarding preparedness for the research journey, support systems influencing preparedness for the research journey and hope for change in the research progress when concerns are addressed. Facilitating preparedness, resilience and research support were the main concepts that grounded the process of the model development.

**Conclusion:**

The successfully developed model provides a reference framework for improving the completion period of postgraduate studies. As the model has not been tested as yet, we recommend future studies to test the validity and reliability of the model in enhancing preparedness and improving the post-graduate throughput rate.

**Contribution:**

The findings advance human capacity development, such as nurses with master’s and PhD qualifications – as inexperienced research professionals.

## Introduction

The throughput rate, or the timely completion of studies by postgraduate students, plays a significant role in the funding models of nursing education institutions (NEIs) (Botha 2018; Cloete & Mouton 2015; Drennan 2008; Eggins 2008; McCulloch 2007; Tanga et al. 2021). Effective supervision and research progress are key factors that contribute to an increased throughput rate (Wilson & Pool 2024). Both the preparedness of postgraduate students and their supervisors have a direct impact on the completion periods of studies (Sá, Santos & Serpa 2021; Van der Laan et al. 2021). Several scholars have explored the issue of prolonged completion times, which negatively affects the throughput rate of NEIs (Cloete et al. 2015; Noel, Wambua & Ssentamu 2021; The Council on Higher Education of South Africa (SA) [CHE] 2022). Among the factors contributing to delays are reduced university funding, insufficient facilities, a lack of supervision expertise, high supervisor turnover and the supervision models employed (Mahlangu 2021; Sá et al. 2021). However, one of the most critical issues identified is the insufficient preparation of both students and supervisors, which further extends the time required for degree completion (Motseke 2016; Van der Laan et al. 2021).

The CHE (2022) report highlighted the national concern regarding delayed postgraduate study completion, with durations often extending beyond 6 years. To understand this issue fully, it is necessary to consider the varying time frames set by universities both nationally and internationally in their research-related policies. Albertyn and Bitzer (2015) point to variations in completion times across institutions worldwide associated with lack of preparedness for postgraduate supervision and studies. Table 1 compares and illustrates the impact of unpreparedness for postgraduate supervision and studies regarding the completion period of master’s and doctoral studies in three different countries (Albertyn & Bitzer 2015; Kumar & Lee 2011).

**TABLE 1:** Comparison of normal prescription with delayed completion of research master’s and doctoral studies in three different countries.

Research studies	Australia and Canada		South Africa	
	Normal prescriptions (years)	Delayed completion	Normal prescriptions (years)	Delayed completion
Full-time doctoral	4	5–10 years and above	5	6 years and above
Part-time doctoral	6	7–10 years and above	6	7 years and above
Full-time master’s	3	4 years and above	3	4 years and above
Part-time master’s	4	5 years and above	4	5 years and above

As illustrated in Table 1, the impact associated with preparedness for postgraduate supervision and studies is a wide variation of prolonged completion period that requires urgent intervention.

Various studies have proposed strategies to address prolonged completion periods, including capacity-building initiatives like research workshops and specialised training programs (Cloete et al. 2015; Noel et al. 2021). However, little is known about how the perspectives of postgraduate nursing students and their supervisors might contribute to reducing the length of postgraduate training. While the South African Department of Higher Education and Training (DHET) introduced the University Capacity Development Programme (UCDP) to support these efforts, its impact on shortening completion times remains minimal (CHE 2022). The UCDP, funded by the DHET, provides financial support to NEIs for activities such as doctoral training, postgraduate retreats, and mentoring programs (CHE 2022). Although the program aims to enhance the capacity of supervisors and students alike, some students have reported inadequate utilisation of these funds for thesis completion, which negatively affects their preparedness for postgraduate studies (CHE 2022). Despite the benefits of the UCDP, such as research training to improve supervisor preparedness, ongoing challenges in postgraduate supervision continue to hinder timely completions (Mahlangu 2021).

The CHE (2022) report emphasises the need for academic departments, including nursing, to establish strong support systems for both students and supervisors to improve their readiness for postgraduate research. Cloete et al. (2015) identify several key qualities necessary for the timely completion of studies, such as the quality of candidates at entry, the quality of supervision, the structure of the postgraduate program, and the quality of the final dissertation or thesis. Postgraduate qualifications hold significant potential for career advancement (CHE 2018; Fourie-Malherbe, Albertyn & Bitzer 2016). However, factors such as inadequate preparation by students and inexperienced supervisors, combined with poor-quality supervision, can prevent students from realising these benefits (Motseke 2016; Van der Laan et al. 2021). Understanding how to better prepare both students and supervisors for the research journey could improve the timely completion of postgraduate studies, leading to enhanced career prospects and sustained funding for NEIs.

### Rationale of the study

The rationale for addressing the delayed completion of postgraduate studies includes among others; working towards the targets of the national development plan of 2030 to increase the number of lecturers with doctoral qualifications in higher education, and address the growing demand for professionals and academics with research expertise in academic and clinical healthcare institutions in SA. Moreover, resolving the issue of delayed research progress could significantly contribute to advancing human capacity development, such as nurses with master’s and PhD qualifications. Well-capacitated inexperienced research professionals have a positive influence on academic and clinical healthcare institutions which includes the employment of more specialised nurses, increased supervisory capacity and potential knowledge exploitation and production.

### Research question

How can post-graduate research nursing students (PGRNS) and inexperienced supervisors be prepared for a joint research journey?

### Aim

To develop a model to facilitate the preparedness of (PGRNS) and research supervisors (RS) for a joint research journey in NEIs.

## Research designs and methods

Theory generation of the qualitative research approach with exploratory and descriptive designs were applied to understand the perceptions of post-graduate students and RSs regarding their preparedness in post-graduate studies. Theory generation proceeded according to the following selected steps from Walker and Avant (2011); Chinn and Kramer (2018).

Step 1: A concept analysis.

Step 2: Placing concepts in relationships.

Step 3: Description of the developed model.

### Development of the model

#### Step 1: A concept analysis

Concept analysis is a technique used to determine the attributes of the concept that can be used to create a conceptual meaning (Chinn & Kramer 2018; Walker & Avant 2019). The population of the study was divided into two groups of participants – postgraduate students and RSs who were purposively selected from Gauteng NEIs that permitted the primary author to conduct interviews. Post-graduate research nursing students were selected based on the reason of them studying post-graduate research studies. Research supervisors were selected because they were the main supervisors for research studies of PGRNSs.

Table 2 indicates the demographic profile of the PGRNSs which suggest that most of the postgraduate studies in South Africa are conducted in a part-time mode of studies, and this is different from international countries, such as Australia, the United Kingdom and Malaysia, which have most PGRNSs pursuing full-time studies (Daniel, Kumar & Omar 2018).

**TABLE 2:** Demographic data of postgraduate research nursing students.

PNS	Gender	Type of qualification	Type of studies	Supervision style	Level of study
PNS1	Female	Masters-coursework	Part-time	Co-supervision	2nd level
PNS2	Female	Masters-coursework	Part-time	Co-supervision	2nd level
PNS3	Female	Masters-coursework	Part-time	Co-supervision	2nd level
PNS4	Female	Masters-coursework	Part-time	One supervisor	2nd level
PNS5	Male	Masters-coursework	Part-time	Co-supervision	2nd level
PNS6	Female	Masters-coursework	Part-time	Co-supervision	3rd level
PNS7	Female	Masters-Research	Part-time	Co-supervision	2nd level
PNS8	Female	Masters-Research	Part-time	Co-supervision	3rd level
PNS9	Female	Doctoral-research	Part-time	Co-supervision	3rd level
PNS10	Female	Doctoral-research	Part-time	Co-supervision	3rd level
PNS11	Female	Doctoral-research	Part-time	Co-supervision	3rd level
PNS12	Female	Doctoral-research	Part-time	Co-supervision	1st level
PNS13	Female	Doctoral-research	Part-time	Co-supervision	1st level
PNS14	Male	Doctoral-research	Part-time	Co-supervision	3rd level
PNS15	Female	Doctoral-research	Part-time	Co-supervision	2nd level
PNS16	Male	Doctoral-research	Part-time	One supervisor	3rd level

**Total**					**16**

PNS, postgraduate nursing student.

Table 3 demonstrates more female RSs than males who participated in the study. It was further observed that there were RSs who supervised more students than others using the co-supervision model.

**TABLE 3:** Demographic data of research supervisors.

Participants	Years of supervision experience	Gender	Successfully completed supervision		Supervision in progress		Model of supervision
			M	D	M	D	
1	16	Female	9	2	3	2	Co-supervision
8	10	Female	36	4	15	6	Co-supervision
5	9	Female	7	0	5	3	Co-supervision
7	8	Female	10	3	5	3	Co-supervision
10	7	Female	5	0	3	2	Co-supervision
2	7	Female	7	1	4	5	Co-supervision
4	6	Female	2	0	3	0	Co-supervision
6	5	Female	2	0	2	0	Co-supervision
3	4	Female	1	0	7	1	Co-supervision
9	4	Male	5	0	2	4	Co-supervision

M, masters; D, doctoral students.

Unstructured interviews were conducted with the participants until data saturation was achieved (Creswell & Creswell 2018; Hennink & Kaiser 2022; Mwita 2022). Student participants were asked to describe their perceptions regarding postgraduate studies, while RS were requested to narrate their supervision perceptions in postgraduate nursing studies. The primary author probed both participants to obtain rich data about postgraduate supervision and studies. Data saturation was achieved after interviewing the 14th student and the 10th supervisor. The primary author continued conducting the interviews in English until the 16 PGRNS and 12 RS. Thematic content analysis was applied to analyse the verbatim transcribed data of participants and four broad themes and sub-themes were generated:

**Theme 1:** Perceptions regarding postgraduate supervision provided or received

Theme 1 highlighted diverse barriers to research studies and the optimum supervision process by both the participating supervisors and PGRNSs. Predominantly, Theme 1 considered student-related barriers, such as a student’s personality and his or her determination, which plays a role in his or her progress. Furthermore, social factors, such as family responsibilities; employment-related factors, such as a high workload and a lack of study leave were also dominant amongst the barriers of research progress. Some participating supervisors reported that they used the co-supervision model for postgraduate studies they viewed the co-supervision model as a learning process for both supervisors and students. Supervisors however acknowledged that co-supervision presents various challenges, such as lack of support from the other supervisors, logistics challenges of meetings for discussion of students’ research projects and at times, conflicts between the two supervisors. Despite Theme 1 being dominated by the barriers that affect the research progress and supervision process, few supervisors and research students reported satisfaction of the study progress.

**Theme 2:** Perceptions regarding preparedness for the research journey

The theme discussion provided evidence that some participating PGRNSs had mixed feelings in terms of preparedness for the research journey, with notable barriers from the research process, However, others felt that supervision was a learning process yet full of challenges. Post-graduate research nursing students reported that most of the challenges, such as a high academic workload, and ill heath during the research process, required resilience and determination to overcome them. While RS perceived challenges such as conflict with colleagues regarding students activities.

**Theme 3:** Support systems influencing preparedness for the research journey

The findings indicated that both groups of participants perceived different support systems on postgraduate supervision that influenced the research progress of students positively and also negatively. The following support systems are among the other systems that can be utilised by both PGRNSs and supervisors during the research process; the centre of academic excellence that provides writing and language-related support; library facilities, which provide literature support or sources of information; provision of research workshops and/or seminars organised by the university and funding of scholarships for students offered by the university and other independent structures, such as the National Research Foundation (NRF).

**Theme 4:** Hope for change in the research progress and concerns to be considered

The students’ participants suggested possible ways of creating positive research supervision within academic institutions based on their perceptions of their postgraduate research supervision. According to the participating PGRNSs positive research supervision will be possible when the necessary academic assistance is available.

Participating PGRNSs perceived barriers to research supervision progress, such as a lack of study leave, workload versus balancing time with family and employment responsibilities, inadequate supervisory processes and infrastructure and difficult research processes. Participating supervisors perceived various barriers to optimum research supervision. It appeared that both groups of participants found it difficult to realise a good-quality supervision process during postgraduate studies because of different barriers emanating from personal, family, and employment situations, as well as academic institutions. Therefore, there is a need to prepare both PGRNSs and supervisors for a joint research journey to minimise the barriers and maximise the support discussed. A conceptual framework was developed using the concept analysis method where the identified four themes grounded the following steps:

*Phase 1: Identification of the main concepts for the model*: The themes assisted the primary author in identifying, isolating and selecting the main concepts of the study, namely, facilitating preparedness, resilience and support that guided the development of the conceptual framework and the model. Concept identification and analysis assisted the primary author in putting concepts in a relationship for the development of the model as guided by the six survey elements of Dickoff, James and Wiendenbach’s (1968).

#### Step 2: Placing concepts in relationships

A relationship statement is a link between two or more concepts in a model (Chinn & Kramar 2018). Walker and Avant (2019) further argue that assessing the relationship of concepts in terms of their type, and symmetry is crucial to determine their function within the envisioned model for development. This step assisted the primary author in classifying the three concepts for the development of conceptual frameworks of preparedness.

*Phase 2: Classification of the concepts for the development of conceptual frameworks*: Table 4 illustrates how the classification of concepts for the conceptual framework was achieved as guided by Dickoff et al. (1968) survey list.

**TABLE 4:** Survey list of the model.

Survey list element	Description	Application to the model
Agent	The person who performs the activity	All research supervisors, Head of the Nursing Department
Recipient	People who receive the activity or benefits from the agent’s action.	Postgraduate research nursing students registered and inexperienced research supervisors (IRSs) at NEIs
Context	The context where the agent performs an activity, and the recipient receives the result of the action	The nursing educational environment
Dynamics	Power sources for the activity, whether chemical, physical, biological, mechanical or psychological	The resilience of both supervisors and PGRNSs as well as the support provided to both groups to facilitate the research journey.
Procedure	The path, steps and rubrics – or generally the pattern of the activity	The procedure clarifies the steps to facilitate the preparedness of both groups for the joint research journey.
Terminus	The endpoint or accomplishment of the activity	Adequately prepared IRS and PGRNSs to facilitate the joint research journey to ensure timeous completion of postgraduate studies.

NEIs, nursing education institutions; PGRNSs, post-graduate research nursing students.

*Phase 3: Clarification, definition and use of the main concepts*: As recommended by Chinn and Kramar (2018), various sources, including the available literature, people’s perceptions about the concepts, a thesaurus and dictionaries were utilised to isolate concepts’ attributes and obtain clarity, definitions and uses of the concepts. Table 5 illustrates the summary of essential and related attributes for ‘preparedness’, ‘resilience’ ‘support’ of postgraduate students and supervisors. These essential and related attributes were synthesised to construct the definitions of the concepts used in the current paper for the development of the model.

**TABLE 5:** Essential and related attributes of main concepts.

Preparedness		Resilience		Support	
Essential attributes	Related attributes	Essential attributes	Related attributes	Essential attributes	Related attributes
Risk assessment	Awareness Eagerness Willingness	Ability to control	Creativity Problem-orientated skills Realistic planning	Caring	Assistance to make study progress Attention Guidance
Adequate resources	Readiness Possession of adequate knowledge and skills	Character	Perseverance Quick recovery	Mentoring	Administrative guidance Encouragement Increasing independence
Evaluation	Meeting research needs	Inner force	Ability to change Learning to cope Hope	Providing resources	Helping to succeed or to make progress Provision of funding
Implementation	Professional development Training	-	-	-	-
Planning	Coordination Collaboration	-	-	-	-

### Definition of concepts

The following definitions are generated from the perceptions of students and RSs about postgraduate supervision and studies. Concept analysis was conducted as depicted in Table 5 to identify attributes and synthesise the definition of the concepts defined further in the text:

The significant concepts of the model are defined further in the text using the findings of Table 5:

#### Facilitate preparedness

Facilitating preparedness as a concept includes a needs assessment process conducted by experienced research supervisors (ERSs) to PGRNS and inexperienced research supervisors (IRSs) to enable their preparedness for a joint research journey of post-graduate studies. Unpacking this process, more concepts that are assisting towards clarity of the main concepts are now defined further in the text.

*Preparedness*: The concept of ‘preparedness’ refers to the self-awareness of PGRNSs and IRSs about their readiness and willingness to embark on the research journey of post-graduate studies in NEIs.

*Resilience*: Resilience refers to the coping skills of either the PGRNS or IRSs to recover from difficult research circumstances such as lack of a research support system, delayed research progress and failure to meet research deadlines within a nursing environment.

*Research support*: The concept of ‘research support’ refers to a process of providing academic research assistance targeted to eliminate the research hazards for post-graduate research studies in the form of guidance and training or any special assistance such as funding provided by family members, spouses, friends, employers and NEIs aimed at meeting the research needs of PGRNS and IRS in a joint research journey.

*Post-graduate research nursing student*: The PGRNS is a graduate student who embarks on a research journey of advanced research studies with RSs from NEIs.

*Research supervisor*: An ERS is a research expert with more than 6 years of experience in supervision who embarks on a joint research journey with IRS and PGRNS to supervise, provide research guidance and approve the research activities. Inexperienced Research Supervisors refer to less-ERSs with 5 years of supervision experience who still require support and guidance to provide supervision to PGRNS during a joint research journey in NEIs.

*Inexperienced research professionals*: Inexperienced research professionals refer to novice RSs and postgraduate nursing students (M&Ds) who receive support and guidance about supervision during a joint research journey.

*Joint research journey*: A joint research journey refers to an academic journey of working together with PGRNS, IRSs and ERSs to enhance the preparedness to ensure timeous completion of post-graduate studies in NEIs.

### The model to facilitate the preparedness of post-graduate research nursing students and inexperienced research supervisors for the joint research journey

The results are categorised according to the evaluation and description of the developed model.

### Model evaluation

The objective of model evaluation was to obtain the inputs and feedback that contribute to improving the quality of the model for significant impact in post-graduate research nursing supervision. The primary author sent requests to eight experts for participation in the model evaluation via email communication using the following criteria; the expert has research knowledge, and has either participated in model development or qualitative research. However, only six experts participated in the evaluation of the model. One expert was an international authority while the rest were from SA. All six experts were nurse educators of different academic ranks who had developed a model during their research activities. The experts rated the section questions using a score from 1 to 5 (1 being less & 5 being more important) based on five criteria (Clarity, simplicity, generality, usefulness/utility and significance of the model) recommended by Chinn and Kramer (2018).

**FIGURE 1:**
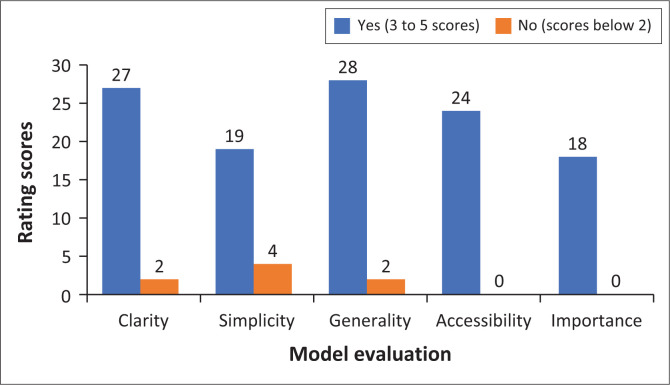
Model evaluation results based on experts’ responses.

The criteria comprised questions rated between 1 and 5, and also a few closed-ended questions (‘yes’ or ‘no’ questions). The experts took almost 3 weeks to complete the evaluation of the model. Figure 1 summarises the findings from six experts who rated the model using the above five criteria, with a total of 30 as maximum scores.

As illustrated earlier in the text, only two sections – accessibility and importance of the model – had a total rating of between 3 and 5 (coded as ‘yes’ in the figure) while the remaining three – clarity, simplicity and generality – had a score of 2 and below (coded as ‘no’ in the figure). Although the three sections (clarity, simplicity and generality) had scores of 2 and below, the majority of experts managed to rate them 15 and above, which the primary author regarded as a good evaluation of the model. The following sections summarise the findings from experts based on the evaluation tool.

### Clarity of the model

**FIGURE 2:**
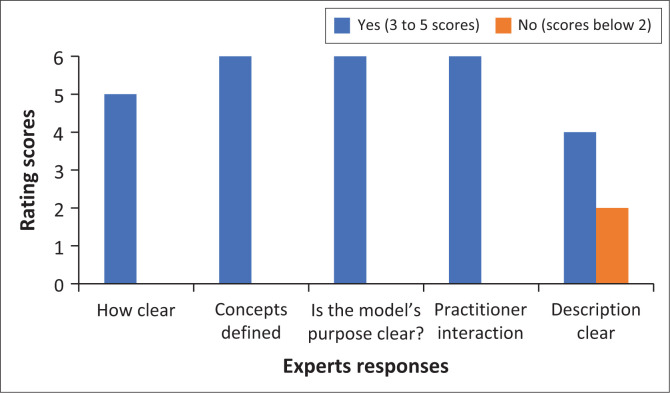
Clarity of the model.

A model should be clear and easily understandable (see Chinn & Kramer 2018). The experts were requested to rate the clarity of the model on a scale of 1 to 5, with 1 being ‘not clear’ and 5 being ‘clear’. This section of the model evaluation comprised six questions, with one of them referring to the purpose of the model. Figure 2 presents the expert responses on the criteria of clarity of the model:

All experts (100%) rated the purpose and concept definition of the model as ‘clear’. In terms of how the nursing personnel could interact with the model, all experts indicated that the interaction of the model was feasible; hence, the rating was 5. As depicted in Figure 2, only two out of six experts indicated that the description was not clear, thus suggesting that experts found the model to be clear. The following are some of the comments made by experts regarding the clarity of the model:

The model is clearly presented as the primary author demonstrated the process, followed when developing the model. The model is clear and well-described in terms of the structure and process of the model … (Expert 5, Female; Expert 3, Female)

The theoretical relationships between and among concepts were well-described. The diagrammatic description of the model is also clear to the reader. The model is quite clear, with all concepts defined, and relationships described and illustrated. (Expert 4, Female)

The model allows for understanding the model with ease and in a very short time. The concept definitions are clear and fitting. (Expert 5, Male)

The experts praised the model for its clear presentation, logical structure, well-defined concepts and easily understood relationships.

### Simplicity of the model

**FIGURE 3:**
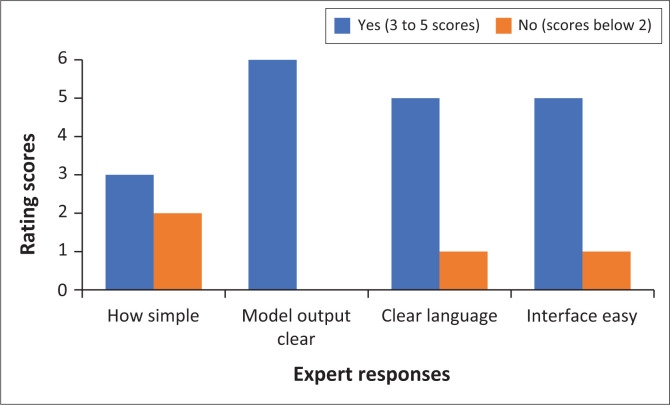
Simplicity of the model.

‘Simplicity’ refers to the model being simple, avoiding unnecessary complexity (see Chinn & Kramer 2018). Experts were asked to evaluate the simplicity of the model by answering only four questions, with one question rated on a scale of 1 to 5, with 1 being ‘not simple’ and 5 being ‘simple to understand’. As depicted in Figure 3, the majority of the experts (83%) rated the model as ‘simple’, based on the model output, interface and language, which scored 5, and only three out of 6 experts rated simplicity of the model 2 and below.

To support the ratings, the following comments have been extracted from some of the experts’ comments regarding the simplicity of the model:

The model is quite simple. All, and only, key concepts are illustrated. A lot of other additional information is conveyed as simple colour codes that were also defined. (Expert 2, Female)

[*T*]he model is clear and every primary author or theory-generative expert or educator will understand the model with ease. (Expert 2, Female)

The model is well-organised in the process and the product. The colours are applicable and well-defined for the model. (Expert 5, Male)

Good colour choices and layout, and the explanation of the colours chosen are clear, well done. (Expert 1, Male)

All experts (100%) therefore found the model to be simple to understand without any challenges expected to be experienced by nurses and other professionals. Thus suggesting that, the model’s clarity, simplicity, effective use of colour, and good organisation, were easy to understand and use by the experts.

### The generality of the model

**FIGURE 4:**
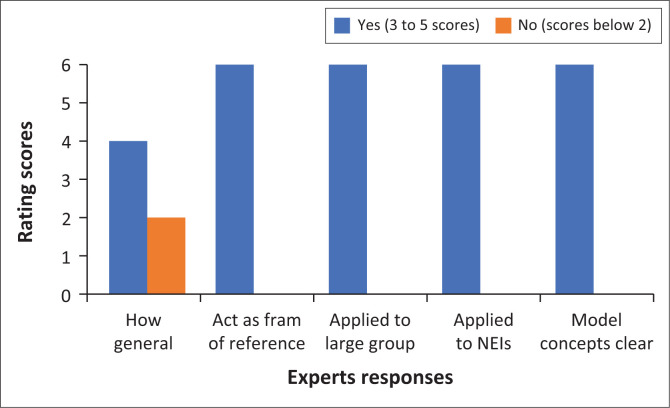
Generality of the model.

The generality of a model refers to its breadth of scope and purpose (see Chinn & Kramer 2018). The generality of the model that was developed refers to the broad scope that is applicable across different patient populations, healthcare settings and nursing contexts. Figure 4 demonstrates that only one expert rated generality 2 and below, with other experts rating it at 5. These ratings demonstrate that the model can be used and applied in various contexts where mentoring and enhancement of research skills play a role.

To support the rating by the experts, the primary author extracted the following comments from the evaluation tool under the generality of the model:

[*T*]he model applies to a variety of contexts and it could be applied in various disciplines as it ensures the preparation of PGRNSs and their IRSs. (Expert 3, Female)

The model stipulates the nursing education environment. (Expert 4, Female)

Seemingly, the experts shared the same sentiments that the model is relevant to the nursing education context, and other disciplines.

### Accessibility of the model

**FIGURE 5:**
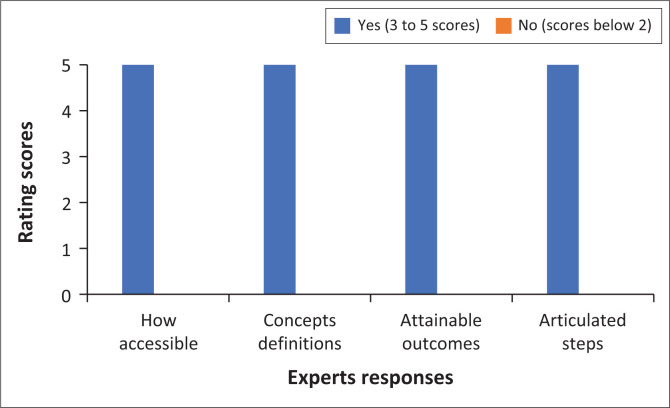
Accessibility of the model.

Chinn and Kramer (2018) emphasise that a model should be accessible for empirical testing and validation. In the current study, the model evaluation consisted of four questions, and the primary author requested the experts to rate the model. Figure 5 demonstrates that all experts rated the accessibility of the model 5.

The following extracts support the fact that the experts found the model to be accessible:

[*A*]ccessibility of this model will be determined by several factors hence I allocated a 3 for this criterion. This will depend on whether the primary author publishes this output in an accessible journal that is international and available to the majority of primary authors of various disciplines. The definitions adequately reflect their meaning to readers. (Expert 1, Male)

The definitions are conceptually well-defined. The steps of the model are aligned with the schematic representation of the model. (Expert 6, Female)

The experts confirmed that the model is accessible because of clear concept definitions and articulated steps for achieving the desired outcomes of preparedness for a joint research journey.

### The importance of the model

**FIGURE 6:**
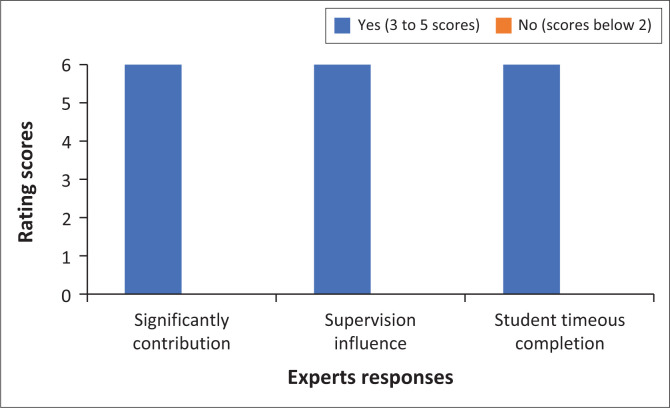
Importance of the model.

According to Chinn and Kramer (2018), a model should address important nursing issues and have practical significance in improving patient care, nursing practice, or healthcare outcomes. As depicted in Figure 6, all experts rated the model as an important contributor to postgraduate studies, the period to completion of studies and research supervision.

A rating of 3 by one expert suggested that the model should be tested or applied to determine its significance within the nursing profession and other disciplines. Below are some quotes to support the rating by the experts regarding the importance of the model to the nursing field.

Learning by discovery always takes time, with a risk of mistakes of varying magnitude and implications. Utilisation of this model can potentially eliminate those. (Expert 3, Female)

This might not be clear at present as the model has not yet been subjected to testing or application. The uniqueness might maybe be based on the fact that the primary author tries to apply the concepts of this model in a unique and new way in guiding supervisors and their postgraduate students. (Expert 1, Male; Expert 2, Female)

The model greatly contributes to the world of nursing research. I enjoyed reading the detail of each step. (Expert 6, Female)

The above comments thus refer to the fact that the model is promising, with the potential to streamline learning and supervision in nursing research, though its effectiveness still needs testing. Although the experts found the model to be clear, simple and important enough to be generalised across nursing and other disciplines, suggestions were made to improve the quality of the model. Some of these suggestions are presented below as expert extracts:

Print the model in colour to enhance accessibility … (Expert 4, Female)

Test or apply the model in real settings to demonstrate its uniqueness and contribution to the nursing discipline … (Expert 4, Male)

Includes the summary of the core research needs under needs assessment phase. (Expert 1, Male)

Well defined, but I would like to see some practical examples of how the model would be implemented effectively in terms of the timelines provided and resources per department. (Expert 3, Male)

Incorporate the issues of mentoring and funding – and consider reducing the long description of the steps. (Expert 2, Female)

I want the candidate and supervisors to include possible software packages as part of the process and to gain knowledge and application of the packages. (Expert 6, Female)

The experts provided a significant feedback about the model which was integrated in the final model presented in this paper. The experts contributed to the technical aspects of the model, including the use of colours, areas for improvement such as the use of arrows, contents to be expanded and consideration for packaging the model as a software for future uses. The expert evaluators found the model to be significant to the nursing environment and appropriate to achieve postgraduate outcomes both in practice and research.

#### Step 3: Description of the model

Walker and Avant (2019) propose the use of the following elements to clarify the scope of the model:

*Purpose of the model*: To provide a theoretical and structural framework of reference to the NEIs that desire to achieve a satisfactory post-graduate throughput rate with optimum supervision in the nursing profession.

*Context*: The context of the model to facilitate the preparedness of PGRNS and IRS for the joint research journey is the nursing education environment within NEIs. As the Department of Nursing Science trains post-graduate studies such as honours, masters and doctoral in various nursing disciplines – the nursing environment is a conducive environment for the model implementation. Lastly, the nursing education environment is the only area where registered PGRNSs meet their RSs for a joint research journey.

*The structure of the model*: The structure of the model is described according to the meaning of the shapes and SmartArt Graphics used in the model, and how the shapes relate to a process of enhancing the preparedness of students and supervisors for a joint research journey.

**FIGURE 7:**
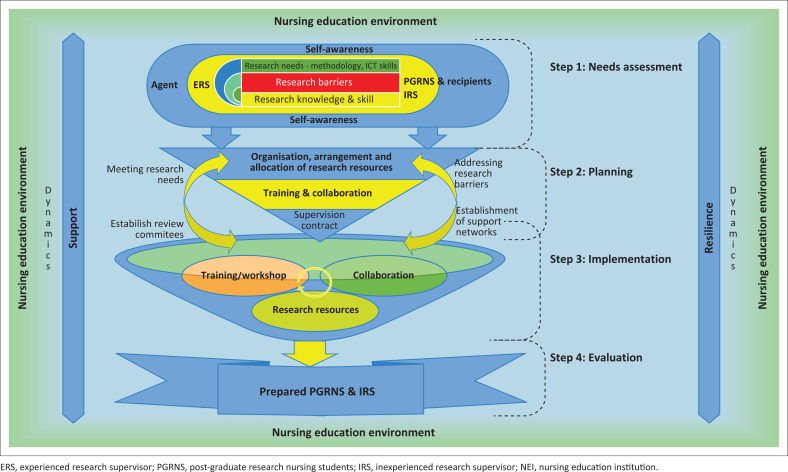
Model to facilitate the preparedness of post-graduate research nursing student and inexperienced research supervisor for their joint research journey.

As depicted in Figure 7, the main feature of the structural description of the model is the oval shape ‘flowchart terminator’ in Yellow and blue as depicted in Step 1. The oval shape ‘flowchart terminator’ in Yellow and blue in Step 1 represent an environment for self-awareness and promoting the student–supervisor relationship. The terminator oval shape refers to the commitment of PGRNS to their post-graduate studies and that of ERS and IRS to support the PGRNS during the joint research journey. Furthermore, the oval shape takes a boardroom-table shape, indicating a conducive environment for the discussion of the research journey activities.

The oval shape terminator in Step 1 contains the target-list shape that separates the model’s agents (ERS) and recipients (PGRNS & IRS). The coloured target list shape with red, green and yellow represents the criteria for self-risk assessment by the PGRNS and IRS during the joint research journey. The criteria are interrelated to assist the PGRNS and IRS in the self-awareness of the research process. Lastly, Step 1 is connected to Step 2 by a yellow descending process arrow, which symbolises the attention given by PGRNS and IRS during their joint research journey.

Step 2 consists of two descending process arrows of SmartArt Graphic coloured yellow, which communicate the planned activities of the joint research journey. The plan activities include signing supervision contracts, research training and collaboration, adequate research resources and addressing research barriers. These are embedded on the two descending yellow arrows, which drop into a funnel shape of Step 3.

Step 3 of the model is depicted by a SmartArt Graphic known as the funnel shape coloured in blue to indicate the implementation process as Step 3. A funnel shape refers to the facilitated process (filtering) of support by an ERS, which merges the research training/workshops, collaboration and research resources utilised in the joint research journey. The filtering process is indicated by three round shapes, which symbolise different research support systems in a funnel shape. The down yellow block arrow connects Step 3 to Step 4 by symbolising outcomes of the filtering process of merged research support systems, such as research training during the joint research journey.

Step 4 of the model contains a curved blue ribbon shape representing the model’s outcomes. The curved down blue ribbon shape is a terminus of the model – timeous completion of post-graduate studies.

Resilience and support as dynamics of the model are symbolised by the blue and brown up-and-down block arrows, which facilitate all the model steps.

*Process of the model*: The process of the model entails the following four steps:

*Step 1: Needs assessment*: Needs assessment refers to a process conducted by PGRNS and IRS before embarking on a joint research journey to identify the research barriers and needs of PGRNS and IRS in a nursing environment. Needs assessment provides an opportunity for facilitators to identify research needs for new services and also to scrutinise the relevancy of existing research services and knowledge (Aven 2016). The model begins with Step 1 (Needs assessment), with the PGRNS and IRS conducting self-awareness, and facilitates awareness regarding existing research knowledge and skills, required research support (to meet the research needs) and research barriers that could delay the progress of the joint research journey. The needs assessment step is achieved with the use of a recommended checklist implemented by the Head of the Department and ERS, which encourages self-awareness of PGRNS and IRS.

*Step 2: Planning*: Planning refers to a process led by the Head of the Department with an ERS to arrange the research resources required during the joint research journey. The two facilitators reflect on the findings of the needs assessment (Step 1), which assists in research resource allocation such as the appointment of an ERS and inexperienced supervisors for supervision and working with the PGRNS in the joint research journey. The appointed ERS organises how the three members (ERSs, IRSs and PGRNSs) will work together during the research journey, which begins with signing the supervision contracts of a joint research journey. Other activities of the planning step include allocating available research resources and organising research training and workshops with various stakeholders (collaborations), which assist in meeting the research needs of PGRNS and IRS.

*Step 3: Implementation*: Implementation involves the Head of Department and ERS’s providing academic research support through guidance, training and resources to postgraduate students (PGRNS) and supervisors (IRS). In this step, the Department of Nursing ensures sufficient research resources are available to remove barriers and meet research needs. The Head of Department and ERS’s facilitate collaborations with institutional research committees and stakeholders to offer workshops, training and access to resources like academic writing centres, ICT platforms and libraries. Additionally, PGRNS may receive support from family, friends and employers to enhance their readiness and progress in the research journey.

*Step 4: Evaluation*: Evaluation is the final step conducted by the Head of the Nursing Department and an ERS to ensure goal-achievement of the research journey. Using a recommended checklist, they assess whether available resources support good research progress and effective supervision. The recommended outcome includes committed students and supervisors ready to leverage the resources for successful research.

### Assumptions of the model

Table 6 illustrates the statements that clarify how the concept ‘facilitate preparedness’ is linked with concepts such as resilience, support, during a joint research journey in a nursing environment.

**TABLE 6:** Assumption statements of the model.

Statements	Model assumptions
Statement 1	The nursing environment enables the PGRNS to embark on a research journey of post-graduate research studies.
Statement 2	The NEIs allow the ERS to embark on a research journey with IRS for post-graduate research studies. Therefore, the ERS guides PGRNS and IRS during a joint research journey within the NEIs. The joint research journey of PGRNS and IRS promotes awareness of their research needs in post-graduate research studies in NEIs.
Statement 3	The identified research needs for PGRNS and IRS assist the ERS in determining the required research support system in a joint journey of post-graduate research studies in NEIs.
Statement 4	The nursing environment allows the ERS, PGRNS and IRS to utilise the available resources during their joint research journey to meet the research needs.
Statement 5	The availability of adequate research resources provided by the NEIs promotes the research progress of the PGRNS and optimum supervision of the IRS, thus meeting their research needs of the joint research journey.
Statement 6	The PGRNS and IRS source various research support from ERS, nursing education institutions, and other stakeholders such as family, spouses, and employers to assist in achieving the outcomes of their joint research journey.
Statement 7	The application of resilience by PGRNS and IRS assists them in achieving the outcomes of the joint research journey of post-graduate research studies.

ERS, experienced research supervisor; PGRNS, post-graduate research nursing students; IRS, inexperienced research supervisor; NEI, nursing education institution.

### Limitations

The current study was limited to a specific context within Gauteng – a province in SA, where only three NEIs permitted participation in the study. The current study findings can therefore not be generalised to other NEIs in SA. The study adopted a theory-generative design and qualitative research approach to answer the research question. This research approach presented limitations in terms of generalising the study findings. Future research studies with a mixed method approach could be applied to enhance generalisation in terms of prolonged completion and supervision of postgraduate research studies. The model is not yet implemented and validated.

## Conclusion

Applying the model within NEIs provides a significant opportunity for enhancing the preparedness of inexperienced research professionals and improving the timeous completion of post-graduate studies. The model provides a framework for preparing PGRNSs and IRSs within NEIs during a joint research journey. The findings significantly contribute to scholarship by providing a frame of reference regarding capacity building aimed at readiness for post-graduate research studies and supervision within NEIs.

### Recommendations

To enhance the model’ s applicability, the authors recommend that the currently developed model be tested in post-graduate studies for future benefits before implementation. The model should be utilised as a reference by the HoD, ERSs, IRSs and PGRNSs who struggle with good research progress and supervision, to improve the timeous completion of postgraduate nursing studies.

### Measures to ensure trustworthiness

The primary author adhered to the four elements to ensure trustworthiness as recommended by Lincoln and Guba (1985, cited in Babbie & Mouton 2009). Confirmability was ensured by using triangulation in data collection. Raw data in field notes and audio recordings were made available to the primary author, independent coder and supervisors only. Furthermore, a meeting between the primary and second authors was held to discuss the themes and sub-themes generated from the interview transcripts by an independent coder. Transferability was ensured by providing a thick, full description of the research methods and designs of the study to enable other professionals to repeat the study. The participants were purposively selected to participate in the study. Dependability was ensured by providing a thick, full description of the research methods and designs of the study and triangulation in data collection (using a digital recorder and field notes). Credibility was ensured by triangulation, audio recordings to record the data during interviews, using an independent coder, debriefing sessions between primary author and supervisor, examination of previous research to frame findings, member checking and using experts in validating the model developed.
